# Automated Detection of Autism Spectrum Disorder Using a Convolutional Neural Network

**DOI:** 10.3389/fnins.2019.01325

**Published:** 2020-01-14

**Authors:** Zeinab Sherkatghanad, Mohammadsadegh Akhondzadeh, Soorena Salari, Mariam Zomorodi-Moghadam, Moloud Abdar, U. Rajendra Acharya, Reza Khosrowabadi, Vahid Salari

**Affiliations:** ^1^Department of Physics, Isfahan University of Technology, Isfahan, Iran; ^2^Department of Electrical and Computer Engineering, Isfahan University of Technology, Isfahan, Iran; ^3^Department of Electrical Engineering, Sharif University of Technology, Tehran, Iran; ^4^Department of Computer Engineering, Ferdowsi University of Mashhad, Mashhad, Iran; ^5^Departement of Computer Science, University of Quebec in Montreal, Montreal, QC, Canada; ^6^Department of Electronics and Computer Engineering, Ngee Ann Polytechnic, Singapore, Singapore; ^7^Department of Biomedical Engineering, School of Science and Technology, Singapore School of Social Sciences, Singapore, Singapore; ^8^International Research Organization for Advanced Science and Technology (IROAST) Kumamoto University, Kumamoto, Japan; ^9^Institute for Cognitive and Brain Sciences, Shahid Beheshti University, Tehran, Iran; ^10^Department of Physical Chemistry, University of the Basque Country UPV/EHU, Bilbao, Spain

**Keywords:** convolutional neural networks, autism spectrum disorder, ABIDE, fMRI, atlas

## Abstract

**Background:** Convolutional neural networks (CNN) have enabled significant progress in speech recognition, image classification, automotive software engineering, and neuroscience. This impressive progress is largely due to a combination of algorithmic breakthroughs, computation resource improvements, and access to a large amount of data.

**Method:** In this paper, we focus on the automated detection of autism spectrum disorder (ASD) using CNN with a brain imaging dataset. We detected ASD patients using most common resting-state functional magnetic resonance imaging (fMRI) data from a multi-site dataset named the Autism Brain Imaging Exchange (ABIDE). The proposed approach was able to classify ASD and control subjects based on the patterns of functional connectivity.

**Results:** Our experimental outcomes indicate that the proposed model is able to detect ASD correctly with an accuracy of 70.22% using the ABIDE I dataset and the CC400 functional parcellation atlas of the brain. Also, the CNN model developed used fewer parameters than the state-of-art techniques and is hence computationally less intensive. Our developed model is ready to be tested with more data and can be used to prescreen ASD patients.

## 1. Introduction

Autism spectrum disorder (ASD), a type of neurological disorder, appears in children between 6 and 17 years of age and affects communication skills and social behavior. ASD affects social interactions and communication and causes repetitive behaviors in patients (Bhat et al., 2014a,b; Huang et al., 2019). According to the WHO, ASD affects one child in 160, and these children often present with other conditions like depression, anxiety, and attention deficit hyperactivity disorder (ADHD)^1^. Early diagnosis during childhood is important and can improve the social skills and communication problems of children with ASD and enhance their quality of life. In order to control and treat this disease, an early diagnosis is crucial. One of the most important tasks for diagnosing neurological diseases such as epilepsy, Alzheimer, and autism is to develop a model based on functional or structural region relationships in the brain (Wing, 1997; American Psychiatric Association, 2011; Chen et al., 2011). Hence, functional magnetic resonance imaging (fMRI) is used to study the brain and its structures. It detects correlated fluctuations in the blood oxygen level-dependent (BOLD) signals from the brain regions. The most common data-driven method for autism diagnosis and the investigation of its biomarkers is the autism brain imaging data exchange (ABIDE), which is a collaborative effort involving neuroimaging and phenotypic data obtained from 1,112 individuals (Di Martino et al., 2014). The ABIDE is a worldwide multi-site database consisting of two phases. The first phase (ABIDE I) consists of 1,112 individuals, with 539 ASD patients and 573 others, from 17 sites. The second phase (ABIDE II) has 521 ASD patients and 593 healthy controls and was obtained from 19 sites. The ABIDE I dataset is obtained from 17 international imaging sites and is composed of structural, resting-state fMRI data and phenotypic information.

Recently, many efforts have been made to identify ASD based on deep learning with fMRI (Koyamada et al., 2015; Anirudh and Thiagarajan, 2017; Subbaraju et al., 2017). In Koyamada et al. (2015), a deep neural network (DNN) model was investigated in order to build a subject-transfer decoder. The authors used principal sensitivity analysis (PSA) to construct a decoder for visualizing different features of all individuals in the dataset. Their proposed neural network includes two hidden layers and a softmax output layer, in which the two hidden layers in the middle classify brain activities into seven human categories from 499 subjects.

It has been shown that ASD disrupts the functional connectivity between the multiple brain regions that affect global brain networks. Therefore, the main goal of many researchers in this area is to classify ASD and control subjects based on the neural patterns of functional connectivity (Bourgeron, 2009; Anderson et al., 2011; Mennes et al., 2011; Schipul et al., 2011; Nielsen et al., 2013; von dem Hagen et al., 2013; Plitt et al., 2015; Dvornek et al., 2017; Parisot et al., 2017, 2018; Aghdam et al., 2018; Xing et al., 2018; Kazeminejad and Sotero, 2019; Sharif and Khan, 2019) and improve the accuracy of classification. For example, Nielsen et al. (2013) achieved 60% classification accuracy, and Abraham et al. (2017) obtained 67% accuracy in classifying ASD and control subjects. Heinsfeld et al. (2018) applied deep learning algorithms to identify ASD patients and improved the accuracy, reaching 70%. They employed two stacked denoising autoencoders to extract a lower-dimensional version of the ABIDE I dataset and also identified the areas of the brain that played the most important role in differentiating ASD from typical controls (TC). The volumetric convolutional neural network (CNN) model, which is considered as the full-resolution 3D spatial structure of resting-state functional MRI data, is investigated in Khosla et al. (2018).

In recent years, the use of CNN has attracted a lot of attention in the field of classification and representation learning. CNNs are powerful classifiers with high accuracies in many applications with many free parameters. Also, CNN models have higher accuracy for feature extraction and can handle many free parameters. The CNN model includes different parts such as an activation function, convolutional layers, fully connected layers, normalization layers, and pooling layers.

The CNN technique has the ability to interpret brain biomarkers in ASD patients using fMRI. The ASD biomarkers play an important role in early diagnosis and treatment (Li et al., 2018b). Li et al. (2018a) proposed multi-channel convolutional neural networks based on a patch-level data-expanding method to diagnose early biomarkers of ASD. Choi (2017), multivariate and high dimensional data are reduced to two-dimensional features, and the functional connectivity pattern associated with ASD is investigated by using a variational autoencoder (VAE).

The stereotypical motor movements (SMM) in autism patients are body rocking and complex hand movements, which will affect learning and social skills. The CNN is used to learn different features from multi-sensor accelerometer signals of SMM (Rad et al., 2015). A fully automated brain tumor segmentation method using CNN was proposed in Havaei et al. (2017).

The purpose of the present study is to investigate the performance of a CNN in classifying ASD and control subjects. We used the fMRI data represented by a multi-site database known as ABIDE I. The ABIDE I data have been preprocessed by the Preprocessed Connectomes Project (PAC). We improved the previously reported results and obtained 70.2% accuracy in the distinction of ASD from control subjects. The performance of the developed model is evaluated using three supervised methods, namely SVM (support vector machine), KNN (K-nearest neighbors), and RF (random forest) classifiers on the preprocessed ABIDE I dataset. Our results show that the average accuracy values after optimization or hyperparameter tuning for SVM, KNN, and RF are 69, 62, and 60%, respectively. Therefore, the proposed CNN model outperformed these machine learning methods. It has been shown that having a CNN model with fewer parameters is very important and leads to less overhead for the new models (Iandola et al., 2016). Our developed model has obtained high accuracy and was also able to train with fewer parameters, which reduces the computation time. An autoencoder has been used to diagnose schizophrenia (Zeng et al., 2018). Functional connectivity MRI data from multiple sites have been used for classification. The authors obtained an accuracy of 85% for multi-site pooling classification and 81% for leave-site-out transfer classification. In this approach, each time, one site out of 17 sites was used as a test and the rest were used for training. The results show that the sites named the Kennedy Krieger Institute, Baltimore (KKI), San Diego State University (SDSU), and University of Utah School of Medicine (USM) achieved higher accuracies than other sites.

The rest of the paper is organized as follows. The details of the ABIDE I dataset, the data preprocessing, and the development of the new CNN model are provided in section 2. In section 3, visual representations of the most important brain areas are presented. Section 4 shows the detailed results of analysis, and finally, the results are discussed in section 5.

## 2. Materials and Methods

### 2.1. Materials

In this work, we used the first phase of resting-state fMRI data from the multi-site ABIDE I. ABIDE I is a consortium of collected resting-state fMRIs from 17 international imaging sites and matched controls that is provided for scientific research. Each site in the ABIDE I dataset uses different parameters and protocols. The fMRI protocol has been used as the imaging protocol at all of the sites. In this work, brain volume is represented by small cubic elements named voxels. The inclusion criteria for sites was having at least 20 subjects meeting other criteria for inclusion like successful preprocessing with manual visual inspection of normalization to MNI space of MPRAGE. The autism diagnostic observation tool and autism diagnostic interview-revised were used for ASD diagnosis or typical control confirmation in the majority of the sites. These types of data increase understanding of the neural bases of ASD. Resting-state fMRI is based on neural measurements of functional connectivity between multiple brain regions. This functional connectivity is calculated by the correlation of the average time series from the regions of interest (ROI). Fluctuations in blood oxygenation lead to low-frequency fluctuation correlations in resting-state fMRI, which gives the connectivity matrix. In the present study, we used the datasets from 505 ASD patients and 530 typical controls. These datasets contain T1 structural brain images, fMRI images, and phenotypic information relating to different patients. The phenotypic information is classified based on sex, age, and autism diagnostic observation schedule (ADOS) score for ASD subjects and mean framewise displacement (FD) quality, which is a measure of subject head motion^2^. The distributions of sex and average age at different sites for typical control (TC) and ASD patients are summarized in Table 1.

**Table 1:** The distribution of sex and average age at different sites for typical control (TC) and ASD classes.

		**TC**			**ASD**		
**Site**	**Abbreviation**	**Average age**	**Sex**		**Average age**	**Sex**	
			**Male**	**Female**		**Male**	**Female**
CALTECH	California Institute	28	14	4	27.4	15	4
	of Technology						
CMU	Carnegie Mellon	26.8	10	3	26.4	11	3
	University						
KKI	Kennedy Krieger	10	20	8	10	16	4
	Institute, Baltimore						
LEUVEN	University of Leuven	18.2	29	5	17.8	26	3
MAX MUN	Ludwig Maximilians	24.6	27	1	26.1	21	3
	University, Munich						
NYU	NYU Langone	15.7	74	26	14.7	65	10
	Medical						
	Center, New York						
OHSU	Oregon Health	10.1	14	0	11.4	12	0
	and Science						
	University						
OLIN	Olin, Institute of	16.7	13	2	16.5	16	3
	Living,						
	Hartford Hospital						
PITT	University of	18.9	23	4	19	25	4
	Pittsburgh						
	School of Medicine						
SBL	Social Brain Lab BCN	33.7	15	0	35	15	0
	NIC UMC Groningen						
	and Netherlands						
	Institute for						
	Neurosciences						
SDSU	San Diego State	14.2	16	6	14.7	13	1
	University						
STANFORD	Stanford University	10	16	4	10	15	4
TRINITY	Trinity Center	17.1	25	0	16.8	22	0
	for Health Sciences						
UCLA	University of California,	13	38	6	13	48	6
	Los Angeles						
UM	University of Michigan	14.8	56	18	13.2	57	9
USM	University of Utah	21.3	25	0	23.5	46	0
	School of Medicine						
YALE	Child Study Center,	12.7	20	8	12.7	20	8
	Yale University						

### 2.2. Data Preprocessing of the ABIDE I Dataset

The Preprocessed Connectomes Project (PCP) is a publicly available preprocessed version of data from both the 1,000 Functional Connectomes Project (FCP) and the International Neuroimaging Data-Sharing Initiative (INDI)^3^. We used data from the FCP using the configurable pipeline, the Analysis of Connectomes (CPAC). After the preprocessing, we obtained 871 quality MRI images with phenotypic information. The preprocessing step included slice timing correction, correction for motion, and normalization of voxel intensity. Nuisance regression was employed to delete the signal fluctuations caused by head motion, respiration, cardiac pulsation, and scanner drift. The signal fluctuation was modeled using 24 motion parameters for head motion, a quadratic and linear term for scanner drift, and CompCor with five principal components for physiological noise (Friston et al., 1995; Fox et al., 2005; Lund et al., 2005; Behzadi et al., 2007). Bandpass filtering (0.01–10 Hz) was used in our analysis.

We used the CC400 functional parcellation atlas of the brain throughout our study. In this atlas, a brain connectivity matrix is constructed for the average time series of the ROI, partitioned into 400 regions. There are many different parameters in MRI imaging, including voxel size, flip angle, TR, TE, and T1. Table 2 summarizes the different parameters in structural MRI imaging for each site in ABIDEI.

**Table 2:** Different parameters in structural MRI imaging for each site in ABIDE I.

	**Voxel size (mm^3^)**	**Flip angle (deg)**	**TR (ms)**	**TE (ms)**	**T1 (ms)**
CALTECH	1	10	1,590	2.73	800
CMU	1	8	1,870	2.48	1,100
KKI	1	8	8	3.7	843
LEUVEN	0.98 × 0.98 × 1.2	8	9.6	4.6	885.145
MAX MUN	1	9	1,800	3.06	900
NYU	1.3 × 1.3	7	2,530	3.25	1,100
OHSU	1	10	2,300	3.58	900
OLIN	1	8	2,500	2.74	900
PITT	1.1 × 1.1 × 1.1	7	2,100	3.93	1,000
SBL	1	8	9	3.5	1,000
SDSU	1	45	11.08	4.3	NA
STANFORD	0.86 × 1.5 × 0.86	15	8.4	1.8	NA
TRINITY	1	8	8.5	3.9	1060.17
UCLA	1 × 1 × 1.2	9	2,300	2.84	853
UM	1.2 × 1 × 1	15	250	1.8	500
USM	1 × 1 × 1.2	9	2,300	2.91	900
YALE	1	9	1,230	1.73	624

In the following, we will describe our proposed CNN architecture in detail.

### 2.3. Network Architecture

In this work, we obtained connectomes or functional connectivity matrices for the detection of ASD classes. This symmetric matrix shows the correlation between the mean values of the time series obtained from an ROI. Each cell in the matrix contains a Pearson correlation coefficient, and each row is the representation of the ROI.

**Figure 1:**
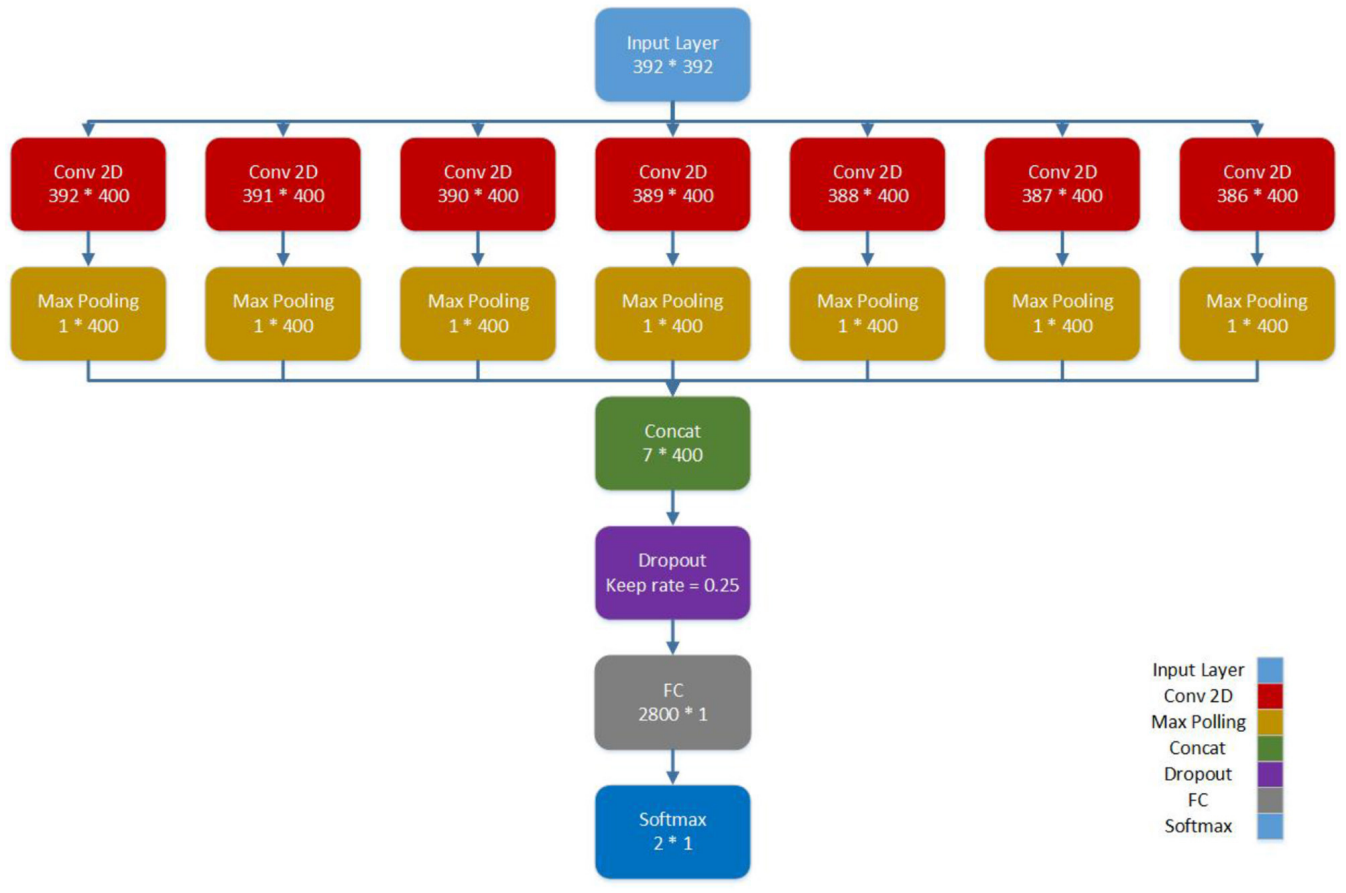
Proposed CNN architecture for automated detection of ASD.

The Pearson correlation coefficient (ranges from −1 to 1) is a correlation index between two areas of the brain regions, with 1 representing high correlation between the two areas of the brain and vice versa. Thus, a 392 × 392 matrix is found in the CC400 functional parcellation atlas for each subject, which represents the co-activation correlations of 392 brain areas. By considering each row as the representation of a brain region, we propose a CNN architecture for connectomic data. We used a CNN architecture with one convolutional layer, interspersed within max-pooling followed by densely connected layers (Please see Figure 1). The functional connectivity matrices between pairs of ROI are fed as input to convolutional layers. Our final CNN model is as follows: 1 fully connected hidden layer and each linear layer followed by a tanh activation function. The parallel filters with dimensions from 1 × 392 to 7 × 392 act on rows representing the brain regions. Thus, we take into account 400 filters of length 1 and width 392–400 filters of length 7 and width 392. In this condition, the sizes of the weights are equal to the representation matrix in the convolutional neural network. The hidden layer followed by max-pooling is used to reduce the number of features and avoid the overfitting problem. After the max-pooling layer, a dropout regularization keeps only 25% of the nodes for training. Finally, the output node is concated and fully connected to a dense layer, which is subsequently used for classification. Also, the model is trained for 300 epochs with a batch size of 32, and the learning rate is set to 0.005. The model as shown in Figure 1 is developed using a 10-fold cross-validation strategy.

The proposed CNN model does not include feed-forward convolution. We employed concatenation of several convolution layers, and the whole result set obtained is passed to the multilayer perceptron (MLP) to complete the classification. In other words, each convolution layer has a specific meaning. For example, when the filter size is 1 × 392, the connection of each area with other areas will be considered, whereas when the filter size is increased to 7 × 392, the connection of 7 areas near each other with other areas will be seen. We combined these outputs to obtain the final output, which is ensemble learning from the convolution layers.

In the next section, we investigate the features that have the most contribution in ASD classification using a visualization method for our proposed CNN model.

## 3. Visualization of Important Areas

**Figure 2:**
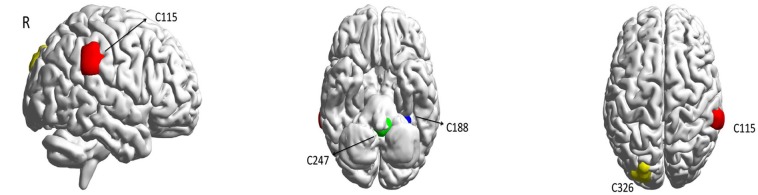
The most important ROIs for ASD classification in the prediction model according to the saliency map. We consider Red, Blue, Green, and Yellow areas corresponding to (61.9; −36.3; 34.4), (−27.6; −40.2; −17.6), (−2.1; −43.0; −40.7), (−22.5; −85.5; 31.0), respectively.

Now, we are interested in visualizing the brain areas that are significant in the classification of ASD and control patients in the ABIDE I dataset. The field of computer vision has enabled vast progress for the visualization of CNN models. In neuroimaging, this technique provides the ability to gain more insights into biomarkers, which are important in early diagnosis and treatment. By using the visualization of image classification models learned via deep Convolutional Networks or ConvNet, we are able to reveal the important ROIs that play important roles in the classification (Simonyan et al., 2013). We obtained the important ROIs for ASD-detection using our model with saliency technique (Figure 2). This approach is based on computing the gradient of the class score with respect to the input image and calculating the class saliency map. In other words, we evaluated the gradient of the output category with respect to the input image:

(1) $$\begin{array}{l} \frac{\partial output}{\partial input} \end{array}$$

Here, output indicates output category, and input is related to input image. The positive ratio indicates that a small change in the input image pixel leads to an increase in the output. Thus, we can obtain salient images of brain areas that play important roles in ASD detection.

We observed that four brain areas are significant in the diagnosis of ASD subjects for the CC400 functional parcellation atlas of the brain. These areas are named as C115, C188, C247, and C326, with the centers of mass equal to (61.9; −36.3; 34.4), (−27.6; −40.2; −17.6), (−2.1; −43.0; −40.7), and (−22.5; −85.5; 31.0), respectively (Table 3).

**Table 3:** Important areas for ASD classification in prediction mode.

**Color**	**Red**	**Blue**	**Green**	**Yellow**
ROI number	C115	C188	C247	C326
Center of mass	(61.9; −36.3; 34.4)	(−27.6; −40.2; −17.6)	(−2.1; −43.0; −40.7)	(−22.5; −85.5; 31.0)

Our results show that the right supramarginal gyrus, which is considered to preserve self-other distinction during empathy in ASD patients (Hoffmann et al., 2015), seems to play a significant role in the diagnosis of autism. The fusiform gyrus, which is hypoactive in patients with autism (van Kooten et al., 2008), is also emphasized for ASD prediction. Also, the cerebellar vermis is indicated as an important area for the ASD classification, and this was reported to be smaller in autism cases (Kaufmann et al., 2003). In addition, these results support the idea of the disruption of anterior-posterior brain connectivity in ASD, which has been shown in Just (2004), Kana et al. (2009), and Cherkassky et al. (2006).

## 4. Results

Nowadays, CNN is widely used for dataset classification. In this study, we designed a CNN model for automated detection of ASD using the ABIDE I dataset. The preprocessed neuroimaging data from the ABIDE I dataset is used in our experiment. There are 1,112 subjects (539 diagnosed with ASD, and 573 typical controls) in the ABIDE I dataset, reduced to 871 subjects after preprocessing. There is also a phenotype file for this dataset, which includes the automated metrics, specified with the prefix anat finc. Among them, we evaluated the functional metric called mean framewise displacement and removed the outliers where this parameter was over 0.2.

**Figure 3:**
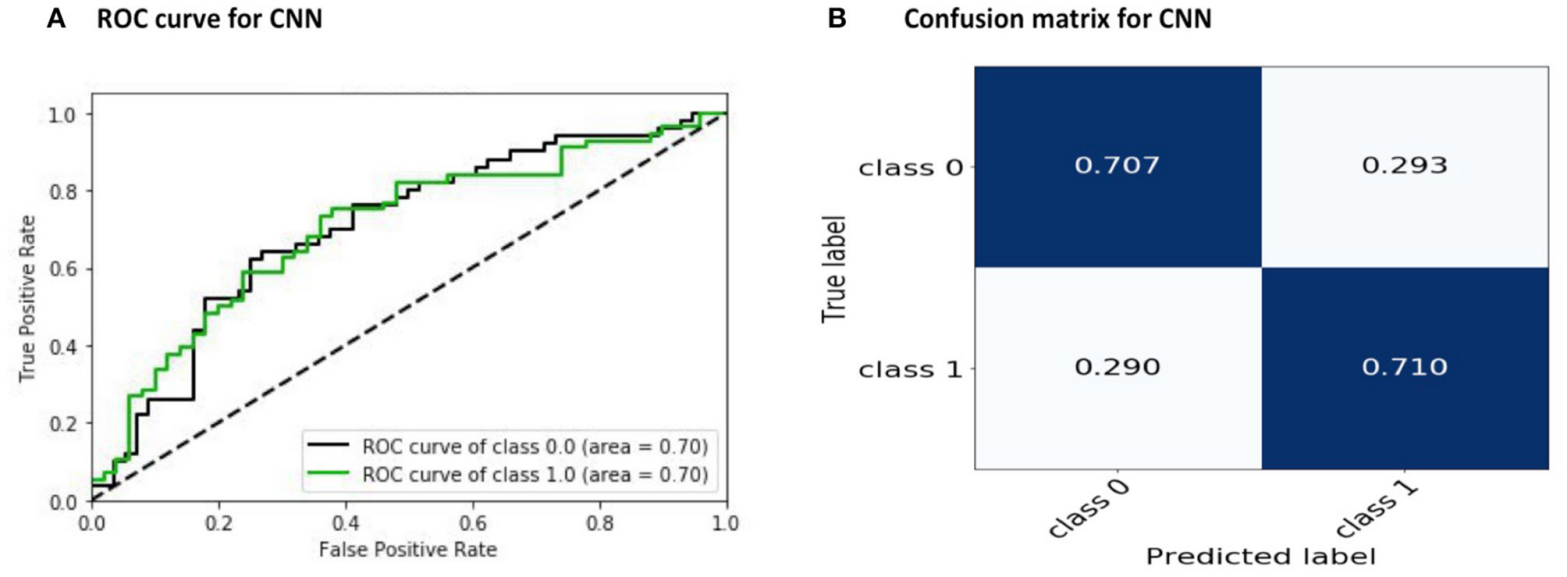
Results of the proposed CNN model: **(A)** ROC and **(B)** convolution matrix. Here, Class 0 and Class 1 indicate the control subjects and ASD patients, respectively.

During training, the learning rate was set at 0.005 with batch sizes of 32 and 400 epochs. The input to the network is a 392 × 392 matrix, where each row represents one of the regions of the brain. In our CNN architecture, we used the 400 filters with sizes from 1 × 392 to 7 × 392. Generally, the width of the filter can be of any size. Here, each row of the connectivity matrix represents the correlation between the corresponding region and the other regions of the brain. Therefore, we considered the width of the filter as the dimension of the corresponding region and equal to the size of each row of the connectivity matrix, which is equal to 392. The length of the filter is its number of rows. Choosing filters of larger sizes did not increase the accuracy of the result. The applied CNN model does not use common feed-forward convolution. In our proposed architecture, we concatenated several convolution layers, and the entire obtained result set was given to the MLP for classification. The filter size of 1 × 392 in the convolution layer means that the connection of each area with other areas will be seen, and the filter size of 4 × 392 means the connection of four areas near each other with other areas will be seen, and at the end, we combine these outputs to get the final output. The execution time for this work was about 12 h and 30 min using 10-fold cross-validation with the NVIDIA Tesla K80 model GPU. We achieved an accuracy of 70.22 %, which is better than the rest of the reported works (Table 4). The receiver operating characteristic curve (ROC) and the confusion matrix for our CNN model are shown in Figure 3.

**Table 4:** Summary of performance values obtained for CNN with 10-fold cross-validation.

**Fold**	**Accuracy**	**Confidence interval**	**Sensitivity**	**Specificity**	**F-score**
1	0.6603	0.0901	0.6250	0.7000	0.6604
2	0.6699	0.0908	0.8889	0.4285	0.7384
3	0.7187	0.0899	0.8113	0.6046	0.7610
4	0.7582	0.0879	0.7755	0.7380	0.7755
5	0.7356	0.0926	0.7659	0.7000	0.7578
6	0.6395	0.1014	0.7826	0.4750	0.6990
7	0.7023	0.0978	0.7777	0.6153	0.7368
8	0.77901	0.0887	0.9318	0.6216	0.8283
9	0.6623	0.1056	0.7380	0.5714	0.7045
10	0.6849	0.1066	0.6500	0.7272	0.6933
Mean	0.7022	0.0855	0.7746	0.6182	0.7355

Thus, to the best of our knowledge, the approach that has been proposed in this paper has obtained the best accuracy so far achieved using the ABIDE I dataset. Table 5 compares the automated detection of TC and ASD classes achieved by different studies using the same database. It can be seen from the comparison table that we have obtained better results compared to the other state-of-art techniques.

**Table 5:** Summary of comparison table for automated detection of TC and ASD classes using the same database.

**References**	**Protocol**	**Best method**	**Performance (%)**		
			**Specificity**	**Sensitivity**	**Accuracy**
Nielsen et al. (2013)	–	Multiple bins and leave- one-out classifier	58.00	62.00	60.00
Parisot et al. (2017)	10-fold CV	Graph Convolutional Networks (GCN)	–	–	69.50
Dvornek et al. (2017)	10-fold CV	LSTM32	–	–	66.80
Parisot et al. (2018)	10-fold CV	Graph Convolutional Networks (GCN)	–	–	70.40
Aghdam et al. (2018)	10-fold CV	Deep belief Network (DBN)	32.96	84.00	65.56
Xing et al. (2018)	5-fold CV	CNN with element-wise filters (CNN-EW)	70.40	66.44	66.88
Kazeminejad and Sotero (2019)	Leave-one-site-out	Deep learning and PCA	65.00	67.00	66.00
Sharif and Khan (2019)	Leave-one-site-out	Multi-Layer Perceptron (MLP) and Feature Selection	–	–	56.26
Abraham et al. (2017)	10-fold CV	SVC-l1 and SVC-l2 Networks	–	–	67.00
Heinsfeld et al. (2018)	10-fold CV	SVM	62.00	68.00	65.00
Heinsfeld et al. (2018)	10-fold CV	Deep Neural Networks (DNN) and transfer learning	63.00	74.00	70.00
Present study	10-fold CV	CNN	61.00	77.00	70.20

We evaluated the performance of SVM (support vector machine), KNN (K-nearest neighbors), and RF (random forest) classifiers on the preprocessed ABIDE I dataset. After optimization (hyperparameter tuning), the average accuracy was found to be 0.69 for SVM, 0.62 for KNN, and 0.6 RF. The results of the three approaches after being trained with 10-fold cross-validation are presented in Table 6. It can be seen that the CNN-based architecture outperformed these ML classifiers in terms of accuracy, specificity, and sensitivity.

**Table 6:** Results of ROC for CNN, SVM, KNN, and RF classifiers before optimization (BO) and after optimization (AO).

	**SVM**		**KNN**		**RF**		**CNN**
	**BO**	**AO**	**BO**	**AO**	**BO**	**AO**	
Mean of accuracy	0.6890	0.6935	0.6142	0.6211	0.5983	0.5994	0.7022
Variance of accuracy	0.0022	0.0011	0.0011	0.0006	0.00062	0.00052	0.0020
Mean of sensitivity	0.7790	0.7459	0.7619	0.7452	0.7474	0.7595	0.7746
Variance of sensitivity	0.0028	0.0026	0.0045	0.004	0.0045	0.005	0.0078
Mean of specificity	0.5855	0.6325	0.4437	0.4784	0.4277	0.4149	0.6182
Variance of specificity	0.0057	0.0049	0.012	0.008	0.0065	0.0030	0.0098
Mean of AUC	0.7533	0.7553	0.6679	0.6724	0.6546	0.6635	0.7486
Variance of AUC	0.0018	0.0017	0.0021	0.0013	0.0005	0.0012	0.0006
Mean of F-score	0.6486	0.6719	0.5279	0.5516	0.5092	0.5015	0.7355

**Figure 4:**
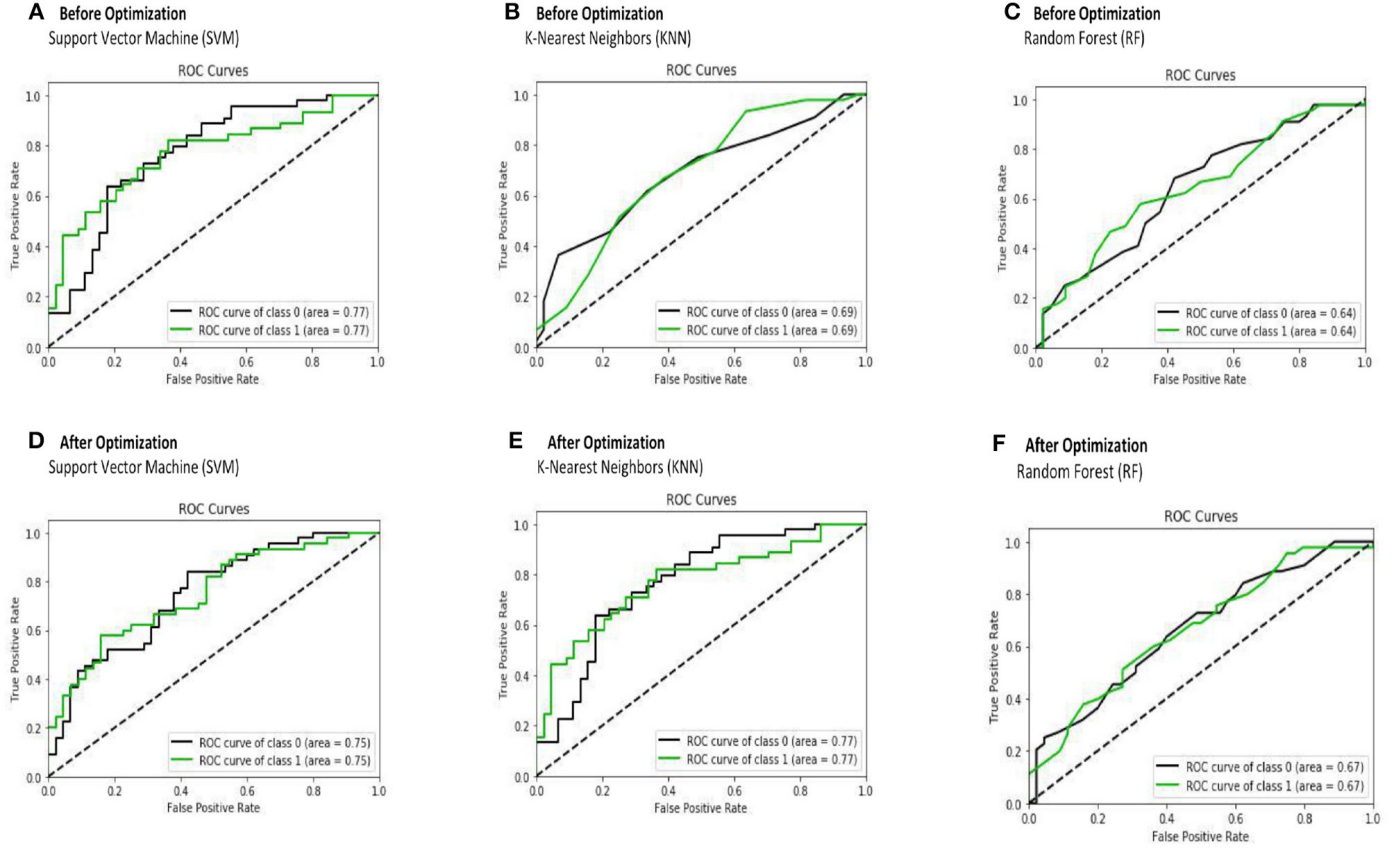
The receiver operating characteristic curve (ROC) is depicted for SVM **(A,D)**, KNN **(B,E)**, and RF **(C,F)** classifiers before and after optimization.

**Figure 5:**
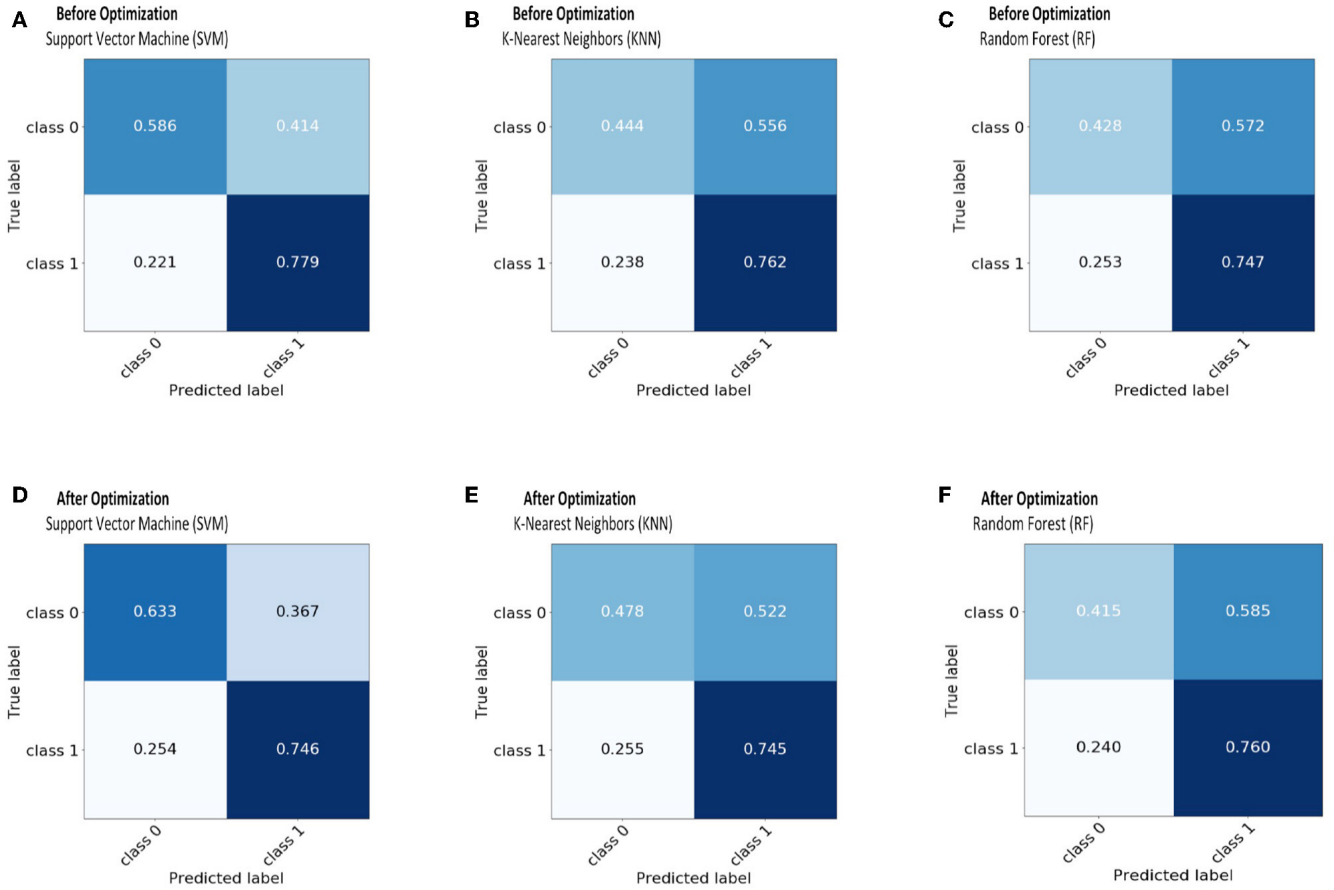
The confusion matrix before and after optimization for classifiers: SVM-**(A,D)**; KNN-**(B,E)**; RF-**(C,F)**.

The receiver operating characteristic curve (ROC) and confusion matrix are used the evaluate the performance of the SVM, KNN, and RF classifiers before and after optimization (hyperparameter tuning), as shown in Figures 4, 5. Before optimization, we used a radial basis function (RBF) kernel with regularization parameter C = 8 for the SVM classifier to obtain the optimum performance. We chose k = 20 for the KNN classifier. We set the max number of features (*max*_*depth*_) and max number of levels in each decision tree (*n*_*estimators*_) as 300 and 100, respectively, for the RF classifier. After optimization, we selected kernals such as “linear,” “rbf,” “poly,” and “sigmoid” for SVM. We employed the grid search method for the KNN classifier and chosen optimization parameters of 4, 8, 12, 16, 20, 24, 28, 32, 36, and 40. The tuning (*max*_*depth*_ and *n*_*estimators*_) of the RF classifier was optimized using the grid search method. In this case, the *max*_*depth*_ values were varied from 120 to 600 with a step size of 60 and *n*_*estimators*_ values were varied from 20 to 180 with a step size of 20. The results show that, by optimizing the tuning parameters, the area under the ROC curve (AUC) will increase and hence, the classification performance is improved. These results are summarized in Table 6.

**Figure 6:**
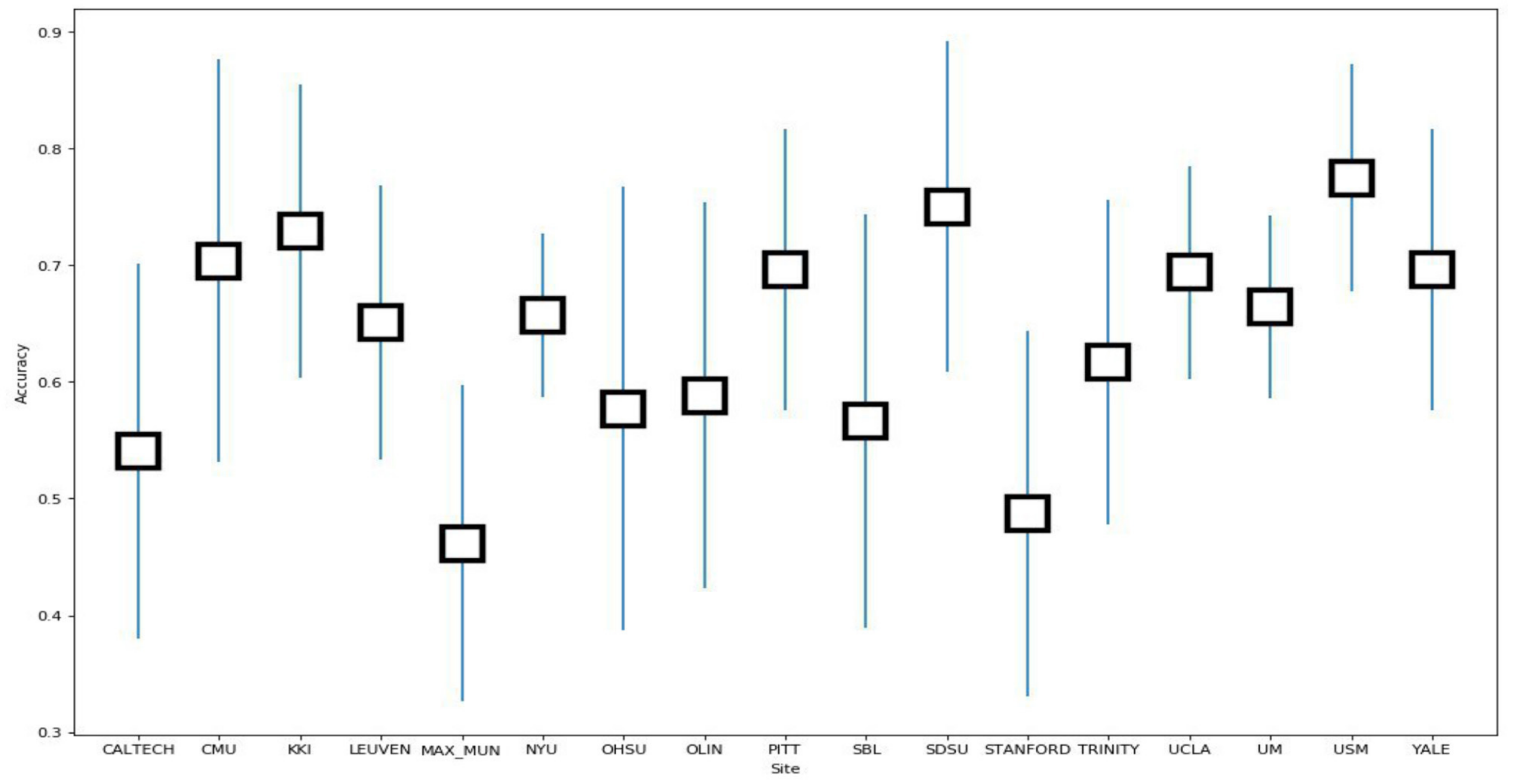
Box plot of accuracy vs. sites.

In order to evaluate the classifier performance for different sites, we used a leave-site-out approach for our proposed CNN, SVM, KNN, and RF classifiers (Heinsfeld et al., 2018). In this method, each site is taken as one fold in the dataset, and we applied a cross-validation approach on the remaining sites instead of different folds. Therefore, each time, one site out of 17 is used to test, and the other sites are used for training. We observe that the sites KKI, SDSU, and USM achieved accuracies of more than 70% as compared to other sites when using our proposed CNN model. The accuracy, confidence interval 95%, specificity, sensitivity, and F-score values for various sites are presented in Table 7 and the accuracy and the confidence interval 95% are depicted in Figure 6. Also, a summary of the performance values obtained for each site using the SVM, KNN, and RF classifiers after optimization are given in Tables 8–10, respectively.

**Table 7:** Summary of performance values obtained for 17 sites using our proposed CNN model.

**Site out**	**Size**	**Accuracy**	**Confidence interval**	**Specificity**	**Sensitivity**	**F-score**
CALTECH	37	0.54	0.16	0.42	0.66	0.58
CMU	27	0.70	0.17	0.71	0.69	0.69
KKI	48	0.72	0.12	0.95	0.57	0.71
LEUVEN	63	0.65	0.12	0.37	0.88	0.73
MAX MUN	52	0.46	0.13	0.45	0.46	0.48
NYU	175	0.65	0.07	0.41	0.84	0.73
OHSU	26	0.57	0.19	0.66	0.5	0.56
OLIN	34	0.58	0.16	0.57	0.6	0.56
PITT	56	0.69	0.12	0.51	0.88	0.73
SBL	30	0.56	0.18	0.4	0.73	0.62
SDSU	36	0.75	0.14	0.64	0.81	0.8
STANFORD	39	0.48	0.16	0.94	0.05	0.09
TRINITY	47	0.61	0.14	0.63	0.6	0.62
UCLA	98	0.69	0.09	0.72	0.65	0.65
UM	140	0.66	0.08	0.95	0.4	0.56
USM	71	0.77	0.09	0.8	0.72	0.69
YALE	56	0.69	0.12	0.82	0.57	0.65
Mean	61	0.63	0.13	0.64	0.62	0.61

**Table 8:** Summary of performance values obtained for 17 sites using the SVM classifier after optimization.

**Site out**	**Accuracy**	**Specificity**	**Sensitivity**	**F-score**
CALTECH	0.70	0.89	0.50	0.62
CMU	0.74	0.71	0.77	0.74
KKI	0.75	0.95	0.61	0.74
LEUVEN	0.63	0.34	0.88	0.72
MAX MUN	0.54	0.54	0.54	0.55
NYU	0.68	0.66	0.69	0.71
OHSU	0.73	0.58	0.86	0.77
OLIN	0.68	0.68	0.67	0.64
PITT	0.70	0.55	0.85	0.73
SBL	0.53	0.40	0.67	0.59
SDSU	0.72	0.64	0.77	0.77
STANFORD	0.61	0.94	0.30	0.44
TRINITY	0.57	0.72	0.44	0.52
UCLA	0.75	0.76	0.75	0.73
UM	0.76	0.80	0.72	0.76
USM	0.79	0.85	0.68	0.69
YALE	0.71	0.75	0.68	0.70
Mean	0.68	0.69	0.67	0.67

**Table 9:** Summary of performance values obtained for 17 sites using the KNN classifier after optimization.

**Site out**	**Accuracy**	**Specificity**	**Sensitivity**	**F-score**
CALTECH	0.54	0.47	0.61	0.56
CMU	0.59	0.35	0.85	0.67
KKI	0.60	0.75	0.50	0.60
LEUVEN	0.62	0.28	0.91	0.72
MAX MUN	0.50	0.50	0.50	0.52
NYU	0.59	0.40	0.74	0.68
OHSU	0.64	0.25	0.64	0.56
OLIN	0.68	0.68	0.66	0.64
PITT	0.58	0.41	0.78	0.65
SBL	0.57	0.33	0.80	0.65
SDSU	0.64	0.36	0.82	0.73
STANFORD	0.51	0.0.58	0.45	0.49
TRINITY	0.60	0.41	0.76	0.67
UCLA	0.63	0.48	0.82	0.67
UM	0.57	0.98	0.20	0.33
USM	0.46	0.20	0.96	0.56
YALE	0.64	0.43	0.86	0.70
Mean	0.58	0.46	0.70	0.61

**Table 10:** Summary of performance values obtained for 17 sites using the RF classifier after optimization.

**Site out**	**Accuracy**	**Specificity**	**Sensitivity**	**F-score**
CALTECH	0.48	0.52	0.44	0.45
CMU	0.66	0.35	1.00	0.74
KKI	0.64	0.90	0.46	0.60
LEUVEN	0.63	0.27	0.94	0.73
MAX MUN	0.56	0.42	0.67	0.62
NYU	0.68	0.50	0.82	0.75
OHSU	0.50	0.58	0.43	0.48
OLIN	0.64	0.63	0.67	0.62
PITT	0.67	0.48	0.89	0.73
SBL	0.60	0.33	0.87	0.68
SDSU	0.63	0.71	0.60	0.67
STANFORD	0.51	0.89	0.15	0.24
TRINITY	0.64	0.59	0.68	0.67
UCLA	0.61	0.46	0.79	0.65
UM	0.67	0.89	0.47	0.60
USM	0.70	0.65	0.80	0.65
YALE	0.66	0.53	0.79	0.70
Mean	0.62	0.57	0.67	0.62

## 5. Discussion and Conclusions

In the present study, we proposed a CNN architecture to identify and classify ASD patients and control subjects. Also, the performance of three supervised learning methods, SVM, KNN, and RF classifiers, on the preprocessed ABIDE I dataset was investigated. The results show that the average accuracy of our model using the test data is 70.2%, meaning that it outperformed the best accuracy obtained on this dataset so far. It has been observed that for the same accuracy, a CNN model with fewer parameters is more efficient and has less overhead for the new models (Iandola et al., 2016). Keeping this in mind, our model is able to train with fewer parameters and achieve an even better accuracy level than the best-performing models. The existing best-known method used a huge number of parameters (19, 961, 200) in its final stage, but our model used 4,398,802 parameters. The authors of Xing et al. (2018) used 1,268,160 parameters and obtained an accuracy of 66.88%, but we achieved an accuracy of 70.20%. Hence, our proposed CNN architecture is able to obtain higher classification performance with fewer parameters, which will reduce the training time. Therefore, our proposed model is less complex and faster as compared to other similar models. Also, we studied each row of the connectivity matrix as the representation of the correlation between the corresponding region and the other regions of the brain in our model.

Thus, we open up the possibility to illustrate the behavior of a region of the brain and corresponding biomarkers by performing a noise correction on each row of the connectivity matrix in future work.

The future recommendations for our proposed model are given below: 1. We have used few images in each class. There is a need to use more data to build a more robust model. 2. The time complexity of the model should be decreased when the whole dataset of all subjects are fed into it. 3. The impact of two features (sex and average age) need to be considered in this study. 4. The performance may improve with balanced data.

## Data Availability Statement

The datasets generated for this study are available on request to the corresponding author.

## Author Contributions

ZS, MAk, SS, MZ-M, and MAb have equal contributions in data preparation, data analysis, and preparing the first draft of the manuscript. UA improved the results and revised the text of the manuscript. RK and VS revised the results and prepared the final version.

### Conflict of Interest

The authors declare that the research was conducted in the absence of any commercial or financial relationships that could be construed as a potential conflict of interest.

## Footnotes

^1^http://www.healthdata.org/gbd

^2^http://preprocessed-connectomes-project.org/abide/quality_assessment.html

^3^http://preprocessed-connectomes-project.org
